# Supplementary material for: Differential occupational risks to healthcare workers from SARS-CoV-2 observed during a prospective observational study
Source: eLife. 2020 Aug 21;9:e60675. doi: 10.7554/eLife.60675 (PMC7486122; doi:10.7554/eLife.60675)
Supplement: Supplementary file 1. — (A) Comparison of serology results by two methods, the Abbott Architect i2000 and Target Discovery Institute ELISA. (B) Univariable and multivariable relationships between risk factors and staff infection with SARS-CoV-2 in 10,032 healthcare workers. (C) Multivariable relationships between risk factors and SARS-CoV-2 IgG positivity in 9956 healthcare workers. (D) Multivariable linear regression, relationship between the percentage of staff with Covid-19 and ward-based Covid-19 pressure and ward type. (E) Rates of self-reported exposure without PPE by staff specialty. [file elife-60675-supp1.docx]

# ­­­Differential occupational risks to healthcare workers from SARS-CoV-2 observed during a prospective observational study

# Supplementary tables

|  | | Abbott Architect CMIA | | |
| --- | --- | --- | --- | --- |
|  |  | **Detected** | **Not detected** | **Not tested** |
| Target Discovery Institute ELISA | **Detected** | 787  (7.8%) | 104  (1.0%) | 14  (0.1%) |
|  | **Not detected** | 114 (1.1%) | 8166  (81.4%) | 121  (1.2%) |
|  | **Not tested** | 50  (0.5%) | 602  (6.0%) | 76  (0.8%) |

**Supplementary File 1A. Comparison of serology results by two methods, the Abbott Architect i2000 and Target Discovery Institute ELISA**. Results shown for 10,034 staff who were tested by serology or PCR with table percentages.

| Variables | | Descriptive | | | | | | Univariable | | | | Multivariable | | | |
| --- | --- | --- | --- | --- | --- | --- | --- | --- | --- | --- | --- | --- | --- | --- | --- |
| Variable group | **Variable** | **Number of staff with this exposure** | **Number of positive staff with this exposure** | **Percentage of exposed staff positive** | **Number of staff without this exposure** | **Number of positive staff without this exposure** | **Percentage of unexposed staff positive** | **Odds ratio** | **Lower 95% CI** | **Upper 95% CI** | **p-value** | **Odds ratio** | **Lower 95% CI** | **Upper 95% CI** | **p-value** |
| Work in Covid-19 patient area | No | 7369 | 631 | 8.6% |  |  |  | 1.00 |  |  |  | 1.00 |  |  |  |
|  | Yes | 1586 | 358 | 22.6% |  |  |  | 3.11 | 2.70 | 3.59 | <0.001 | 2.47 | 1.99 | 3.08 | <0.001 |
|  | Work everywhere | 1079 | 139 | 12.9% |  |  |  | 1.58 | 1.30 | 1.92 | <0.001 | 1.39 | 1.04 | 1.85 | 0.02 |
| Speciality | Other or none | 4346 | 380 | 8.7% |  |  |  | 1.00 |  |  |  | 1.00 |  |  |  |
|  | Anaesthetics | 244 | 17 | 7.0% |  |  |  | 0.78 | 0.47 | 1.29 | 0.34 | 0.74 | 0.42 | 1.30 | 0.29 |
|  | Emergency Medicine | 344 | 41 | 11.9% |  |  |  | 1.41 | 1.00 | 1.99 | 0.05 | 1.03 | 0.68 | 1.58 | 0.88 |
|  | General Surgery, Urology, Plastics, Vascular, Cardiothoracic Surgery | 519 | 65 | 12.5% |  |  |  | 1.49 | 1.13 | 1.98 | 0.005 | 0.98 | 0.67 | 1.42 | 0.90 |
|  | Haematology, Oncology | 333 | 52 | 15.6% |  |  |  | 1.93 | 1.41 | 2.64 | <0.001 | 1.88 | 1.27 | 2.78 | 0.002 |
|  | Infectious Diseases, Respiratory | 209 | 27 | 12.9% |  |  |  | 1.55 | 1.02 | 2.35 | 0.04 | 0.78 | 0.46 | 1.30 | 0.33 |
|  | Intensive Care Medicine | 448 | 44 | 9.8% |  |  |  | 1.14 | 0.82 | 1.58 | 0.45 | 0.44 | 0.28 | 0.69 | <0.001 |
|  | Medicine | 793 | 222 | 28.0% |  |  |  | 4.06 | 3.36 | 4.89 | <0.001 | 1.52 | 1.07 | 2.16 | 0.02 |
|  | Obstetrics and Gynaecology | 250 | 15 | 6.0% |  |  |  | 0.67 | 0.39 | 1.13 | 0.14 | 0.64 | 0.35 | 1.14 | 0.13 |
|  | Ophthalmology, Ear, nose and throat surgery, Maxillofacial surgery | 171 | 13 | 7.6% |  |  |  | 0.86 | 0.48 | 1.53 | 0.60 | 0.87 | 0.46 | 1.63 | 0.67 |
|  | Paediatrics | 588 | 43 | 7.3% |  |  |  | 0.82 | 0.59 | 1.14 | 0.25 | 0.79 | 0.53 | 1.18 | 0.25 |
|  | Radiology | 379 | 37 | 9.8% |  |  |  | 1.13 | 0.79 | 1.61 | 0.50 | 0.99 | 0.60 | 1.63 | 0.98 |
|  | Specialist Medicine | 1087 | 116 | 10.7% |  |  |  | 1.25 | 1.00 | 1.55 | 0.05 | 0.86 | 0.63 | 1.19 | 0.38 |
|  | Trauma and Orthopaedics, Rheumatology | 323 | 56 | 17.3% |  |  |  | 2.19 | 1.61 | 2.97 | <0.001 | 1.86 | 1.26 | 2.74 | 0.002 |
| Role | Administrative Staff | 1218 | 88 | 7.2% |  |  |  | 1.00 |  |  |  | 1.00 |  |  |  |
|  | Biomedical scientist and laboratory staff | 354 | 29 | 8.2% |  |  |  | 1.15 | 0.74 | 1.77 | 0.54 | 1.11 | 0.71 | 1.72 | 0.65 |
|  | Senior Doctor | 704 | 57 | 8.1% |  |  |  | 1.13 | 0.80 | 1.60 | 0.49 | 0.85 | 0.55 | 1.32 | 0.48 |
|  | Junior Doctor | 853 | 113 | 13.2% |  |  |  | 1.96 | 1.46 | 2.63 | <0.001 | 1.08 | 0.72 | 1.60 | 0.72 |
|  | Nurse / Healthcare assistant | 3971 | 562 | 14.2% |  |  |  | 2.12 | 1.67 | 2.68 | <0.001 | 1.35 | 0.96 | 1.89 | 0.08 |
|  | Other | 1452 | 103 | 7.1% |  |  |  | 0.98 | 0.73 | 1.32 | 0.90 | 0.82 | 0.61 | 1.11 | 0.21 |
|  | Other allied health professional | 622 | 43 | 6.9% |  |  |  | 0.95 | 0.65 | 1.39 | 0.81 | 0.79 | 0.50 | 1.26 | 0.32 |
|  | Porter, Domestic cleaner | 323 | 60 | 18.6% |  |  |  | 2.93 | 2.06 | 4.18 | <0.001 | 2.06 | 1.34 | 3.15 | 0.001 |
|  | Physiotherapist, Occupational therapist, Speech and Language therapist | 316 | 47 | 14.9% |  |  |  | 2.24 | 1.54 | 3.27 | <0.001 | 1.56 | 0.99 | 2.47 | 0.06 |
|  | Security, Estates, Catering | 221 | 26 | 11.8% |  |  |  | 1.71 | 1.08 | 2.72 | 0.02 | 1.43 | 0.89 | 2.31 | 0.14 |
| Ethnicity | White | 7237 | 686 | 9.5% |  |  |  | 1.00 |  |  |  | 1.00 |  |  |  |
|  | Asian | 1673 | 281 | 16.8% |  |  |  | 1.93 | 1.66 | 2.24 | <0.001 | 1.51 | 1.28 | 1.77 | <0.001 |
|  | Black | 394 | 71 | 18.0% |  |  |  | 2.10 | 1.60 | 2.75 | <0.001 | 1.66 | 1.25 | 2.21 | <0.001 |
|  | Chinese | 93 | 7 | 7.5% |  |  |  | 0.78 | 0.36 | 1.69 | 0.52 | 0.75 | 0.34 | 1.67 | 0.48 |
|  | Mixed | 242 | 28 | 11.6% |  |  |  | 1.25 | 0.84 | 1.87 | 0.28 | 1.23 | 0.82 | 1.87 | 0.32 |
|  | Not stated | 144 | 17 | 11.8% |  |  |  | 1.28 | 0.77 | 2.13 | 0.35 | 1.16 | 0.68 | 1.97 | 0.58 |
|  | Other | 251 | 38 | 15.1% |  |  |  | 1.70 | 1.20 | 2.43 | 0.003 | 1.26 | 0.87 | 1.83 | 0.23 |
| Gender | Female | 7452 | 812 | 10.9% |  |  |  |  |  |  |  |  |  |  |  |
|  | Male | 2562 | 313 | 12.2% |  |  |  | 1.14 | 0.99 | 1.31 | 0.07 |  |  |  |  |
|  | Prefer not to say | 15 | 2 | 13.3% |  |  |  | 1.26 | 0.28 | 5.58 | 0.76 |  |  |  |  |
|  | Trans | 5 | 1 | 20.0% |  |  |  | 2.04 | 0.23 | 18.31 | 0.52 |  |  |  |  |
| Community exposures | Contact with a known Covid-19 case outside work | 336 | 83 | 24.7% | 9696 | 1043 | 10.8% | 2.72 | 2.11 | 3.52 | <0.001 |  |  |  |  |
|  | Household contact with a known Covid-19 case | 174 | 67 | 38.5% | 9858 | 1059 | 10.7% | 5.20 | 3.81 | 7.11 | <0.001 | 4.82 | 3.45 | 6.73 | <0.001 |
|  | Contact with a possible Covid-19 case outside work | 845 | 122 | 14.4% | 9187 | 1004 | 10.9% | 1.38 | 1.12 | 1.68 | 0.002 |  |  |  |  |
|  | Household contact with a possible Covid-19 case | 565 | 91 | 16.1% | 9467 | 1035 | 10.9% | 1.56 | 1.24 | 1.98 | <0.001 | 1.75 | 1.37 | 2.24 | <0.001 |
|  | Live with another healthcare worker | 2509 | 339 | 13.5% | 7523 | 787 | 10.5% | 1.34 | 1.17 | 1.53 | <0.001 |  |  |  |  |
| Healthcare exposure | Healthcare exposure to known or suspected Covid-19 without PPE | 2165 | 368 | 17.0% | 7867 | 758 | 9.6% | 1.92 | 1.68 | 2.20 | <0.001 | 1.44 | 1.24 | 1.67 | <0.001 |
| Week of testing, week beginning | 20-Apr-2020 | 259 | 30 | 11.6% |  |  |  | 1.00 |  |  |  |  |  |  |  |
|  | 27-Apr-2020 | 774 | 63 | 8.1% |  |  |  | 0.68 | 0.43 | 1.07 | 0.10 |  |  |  |  |
|  | 04-May-2020 | 3502 | 421 | 12.0% |  |  |  | 1.04 | 0.70 | 1.55 | 0.83 |  |  |  |  |
|  | 11-May-2020 | 3031 | 319 | 10.5% |  |  |  | 0.90 | 0.60 | 1.34 | 0.60 |  |  |  |  |
|  | 18-May-2020 | 1493 | 189 | 12.7% |  |  |  | 1.11 | 0.73 | 1.67 | 0.63 |  |  |  |  |
|  | 25-May-2020 | 433 | 46 | 10.6% |  |  |  | 0.91 | 0.56 | 1.48 | 0.70 |  |  |  |  |
|  | 01-Jun-2020 | 542 | 60 | 11.1% |  |  |  | 0.95 | 0.60 | 1.51 | 0.83 |  |  |  |  |
|  |  | **Median - positive staff** | **IQR - positive staff** |  | **Median - positive staff** | **IQR - positive staff** |  |  |  |  |  |  |  |  |  |
| Age, per 10 years |  | 39 | 29-49 |  | 40 | 30-50 |  | 0.95 | 0.90 | 1.00 | 0.04 |  |  |  |  |

**Supplementary File 1B. Univariable and multivariable relationships between risk factors and staff infection with SARS-CoV-2 in 10,032 healthcare workers.** SARS-CoV-2 infection at some time was defined using a composite outcome of SARS-CoV-2 PCR positive and/or SARS-CoV-2 IgG positive. Pairwise interactions were sought between all variables the multivariable model, a single interaction exceeded the p<0.01 screening threshold, representing decreased risk of Covid-19 in Emergency Department staff reporting exposure to a Covid-19 without PPE (p=0.002). However, given the large number of interactions sought and biological implausibility the interaction was omitted from the model presented. The category for 01 June also includes 31 staff tested on 08 June.

| Variables | | Multivariable: serology only outcome | | | |
| --- | --- | --- | --- | --- | --- |
| Variable group | **Variable** | **Odds ratio** | **Lower 95% CI** | **Upper 95% CI** | **p-value** |
| Work in Covid-19 patient area | No |  |  |  |  |
|  | Yes | 2.39 | 1.91 | 3.00 | <0.001 |
|  | Work everywhere | 1.38 | 1.03 | 1.84 | 0.03 |
| Speciality | Other or none |  |  |  |  |
|  | Anaesthetics | 0.70 | 0.39 | 1.25 | 0.23 |
|  | Emergency Medicine | 1.06 | 0.69 | 1.64 | 0.77 |
|  | General Surgery, Urology, Plastics, Vascular, Cardiothoracic Surgery | 0.90 | 0.61 | 1.33 | 0.61 |
|  | Haematology, Oncology | 1.96 | 1.32 | 2.92 | 0.001 |
|  | Infectious Diseases, Respiratory | 0.76 | 0.45 | 1.28 | 0.30 |
|  | Intensive Care Medicine | 0.47 | 0.30 | 0.74 | 0.001 |
|  | Medicine | 1.47 | 1.02 | 2.11 | 0.04 |
|  | Obstetrics and Gynaecology | 0.68 | 0.38 | 1.23 | 0.20 |
|  | Ophthalmology, Ear, nose and throat surgery, Maxillofacial surgery | 0.73 | 0.37 | 1.42 | 0.35 |
|  | Paediatrics | 0.82 | 0.55 | 1.24 | 0.35 |
|  | Radiology | 0.90 | 0.54 | 1.50 | 0.68 |
|  | Specialist Medicine | 0.87 | 0.63 | 1.21 | 0.42 |
|  | Trauma and Orthopaedics, Rheumatology | 1.83 | 1.23 | 2.72 | 0.003 |
| Role | Administrative Staff |  |  |  |  |
|  | Biomedical scientist and laboratory staff | 0.96 | 0.60 | 1.54 | 0.88 |
|  | Senior Doctor | 0.84 | 0.54 | 1.31 | 0.44 |
|  | Junior Doctor | 1.05 | 0.70 | 1.58 | 0.83 |
|  | Nurse / Healthcare assistant | 1.33 | 0.94 | 1.87 | 0.11 |
|  | Other | 0.79 | 0.58 | 1.07 | 0.13 |
|  | Other allied health professional | 0.85 | 0.53 | 1.36 | 0.50 |
|  | Porter, Domestic cleaner | 1.96 | 1.26 | 3.04 | 0.003 |
|  | Physiotherapist, Occupational therapist, Speech and Language therapist | 1.53 | 0.95 | 2.44 | 0.08 |
|  | Security, Estates, Catering | 1.27 | 0.77 | 2.09 | 0.35 |
| Ethnicity | White |  |  |  |  |
|  | Asian | 1.52 | 1.29 | 1.80 | <0.001 |
|  | Black | 1.71 | 1.28 | 2.28 | <0.001 |
|  | Chinese | 0.67 | 0.29 | 1.59 | 0.37 |
|  | Mixed | 1.30 | 0.86 | 1.97 | 0.22 |
|  | Not stated | 1.07 | 0.62 | 1.87 | 0.80 |
|  | Other | 1.30 | 0.89 | 1.89 | 0.18 |
| Gender | Female |  |  |  |  |
|  | Male | 1.19 | 1.01 | 1.40 | 0.03 |
|  | Prefer not to say | 2.30 | 0.49 | 10.82 | 0.29 |
|  | Trans | 3.32 | 0.35 | 31.09 | 0.29 |
| Community exposure | Household contact with a known Covid-19 case | 5.05 | 3.61 | 7.07 | <0.001 |
|  | Household contact with a possible Covid-19 case | 1.69 | 1.31 | 2.18 | <0.001 |
| Healthcare exposure | Healthcare exposure to known or suspected Covid-19 without PPE | 1.47 | 1.26 | 1.70 | <0.001 |

**Supplementary File 1C. Multivariable relationships between risk factors and SARS-CoV-2 IgG positivity in 9956 healthcare workers**. Pairwise interactions were sought between all variables the multivariable model, a single interaction exceeded the p<0.01 screening threshold, representing decreased risk of Covid-19 in Emergency Department staff reporting exposure to a Covid-19 without PPE (p=0.003). However, given the large number of interactions sought and biological implausibility the interaction was omitted from the model presented.

|  | | Coefficient, % (95% CI) | p value |
| --- | --- | --- | --- |
| Intercept | | 32.2 (21.8, 42.5) | <0.001 |
| Covid-19 pressure, per 1% increase | | 0.01 (-0.01, 0.02) | 0.41 |
| Clinical area | Covid-19 general ward | 0.0 |  |
|  | Non Covid-19 area | -21.4 (-32.3, -10.5) | <0.001 |
|  | Covid-19 cohort ward | -16.7 (-39.5, 6.0) | 0.15 |
|  | Covid-19 HDU/ICU | -29.0 (-46.3, -11.7) | 0.002 |

**Supplementary File 1D. Multivariable linear regression, relationship between the percentage of staff with Covid-19 and ward-based Covid-19 pressure and ward type.** See Figure 8B for data fitted. Covid-19 cohort wards admitted only patients with suspected or known Covid-19, whereas Covid-19 general wards were acute medical wards receiving new admissions and acute medical patients initially believed not to have Covid-19. Non Covid-19 areas did not admit suspected Covid-19 patients and any suspected or confirmed Covid-19 patients were transferred off these wards as soon as possible.

| Speciality | Total | Number reporting exposure to a patient with known or suspected Covid-19 without PPE | % |
| --- | --- | --- | --- |
| Medicine | 793 | 333 | 42% |
| Intensive Care Medicine | 448 | 171 | 38% |
| General Surgery, Urology, Plastic, Vascular, Cardiothoracic Surgery | 519 | 173 | 33% |
| Trauma and Orthopaedics, Rheumatology | 323 | 105 | 33% |
| Specialist Medicine | 1087 | 325 | 30% |
| Emergency Medicine | 344 | 92 | 27% |
| Anaesthetics | 244 | 61 | 25% |
| Infectious Diseases/Respiratory | 209 | 51 | 24% |
| Haematology/Oncology | 333 | 75 | 23% |
| Radiology | 378 | 84 | 22% |
| Paediatrics | 250 | 34 | 14% |
| Obstetrics and Gynaecology | 171 | 23 | 13% |
| Ophthalmology, Ear nose and throat, Maxillofacial surgery | 793 | 333 | 42% |
| Other | 448 | 171 | 38% |

**Supplementary File 1E. Rates of self-reported exposure without PPE by staff specialty.**
